# Stunting, Underweight and Overweight in Children Aged 2.0–4.9 Years in Indonesia: Prevalence Trends and Associated Risk Factors

**DOI:** 10.1371/journal.pone.0154756

**Published:** 2016-05-11

**Authors:** Cut Novianti Rachmi, Kingsley E. Agho, Mu Li, Louise Alison Baur

**Affiliations:** 1 Discipline of Paediatrics and Child Health, The Children’s Hospital at Westmead (University of Sydney Clinical School), Sydney, NSW, Australia; 2 School of Science and Health, Western Sydney University-Campbelltown Campus, Sydney, NSW, Australia; 3 Sydney School of Public Health, The University of Sydney, Sydney, NSW, Australia; Northeast Ohio Medical University, UNITED STATES

## Abstract

**Objective:**

The double burden of malnutrition affects many low and middle-income countries. This study aimed to: a) determine temporal trends in the prevalence of underweight, stunting, and at risk of overweight/ overweight or obesity in Indonesian children aged 2.0–4.9 years; and b) examine associated risk factors.

**Design:**

A repeated cross-sectional survey. This is a secondary data analysis of waves 1, 2, 3, and 4 (1993, 1997, 2000, and 2007) of the Indonesian Family Life Survey, which includes 13 out of 27 provinces in Indonesia. Height, weight and BMI were expressed as z-scores (2006 WHO Child Growth Standards). Weight-for-age-z-score <-2 was categorised as underweight, height-for-age-z-score <-2 as stunted, and BMI-z-score >+1, >+2, >+3 as at-risk, overweight and obese, respectively.

**Results:**

There are 938, 913, 939, and 1311 separate children in the 4 waves, respectively. The prevalence of stunting decreased significantly from waves 1 to 4 (from 50.8% to 36.7%), as did the prevalence of underweight (from 34.5% to 21.4%). The prevalence of ‘at-risk’/overweight/obesity increased from 10.3% to 16.5% (all *P*<0.01). Stunting and underweight were related to lower birth weight, being breastfed for 6 months or more, having parents who were underweight or had short stature, and mothers who never attended formal education. Stunting was also higher in rural areas. Being at-risk, or overweight/obese were closely related to being in the youngest age group (2–2·9 years) or male, having parents who were overweight/obese or having fathers with university education.

**Conclusions:**

The double burden of malnutrition occurs in Indonesian children. Development of policy to combine the management of chronic under-nutrition and over-nutrition is required.

## Introduction

Although once considered a problem only of developed countries, the prevalence of obesity has risen during the past 30 years in low and middle-income countries (LMICs).[1–4] At the same time, many LMICs are still dealing with the prevalent public health issue of under nutrition, a situation often described as the “double burden of malnutrition”. This “double burden” may occur in the same country, city or household (mother/ child pair), or in the same individual at different stages of his or her life.[5–14]

In a 2013 Indonesian national survey, Riset Kesehatan Dasar (Riskesdas), the prevalence of underweight in children under 5 years was reported as 19.6%, that of stunting as 37.2%, and that of combined overweight and obesity as 11.9%. That survey also documented the prevalence of combined stunting and overweight/obesity in this age group as 6.8%, a higher prevalence than that of children who were of healthy height but who were overweight or obese (5.1%).[15] While such data are undoubtedly important, the inclusion of children aged less than two years, the period of life when stunting is developing,[16] may make interpretation of the results more difficult. The 2006 WHO Growth Standard also emphasises the use of BMI-for-age as the index of weight relative to height starting at 2 years of age.[17] To our knowledge, there has been no Indonesian report that has reported the trends in prevalence of underweight, stunting and overweight in children aged 2.0–4.9 years, along with the associated risk factors. A better understanding of the double burden of malnutrition in Indonesia, especially in early childhood, would aid decision-making about potential strategies to tackle the problem.

Thus, the primary aim of this study was to determine the temporal trends in the prevalence of underweight, stunting, and at risk of overweight/ overweight or obesity in Indonesian children aged 2.0–4.9 years at four different time points: 1993, 1997, 2000, and 2007. The secondary aim was to examine associated risk factors.

## Methods

### Indonesian Family Life Survey

#### The survey and data collection

We performed a secondary analysis of data collected in 1993 (wave 1), 1997 (wave 2), 2000 (wave 3), and 2007 (wave 4) from the Indonesian Family Life Survey (IFLS). The IFLS is a longitudinal survey of a stratified random sample of households involving both questionnaires and anthropometric measurements, that is representative of 83% of the Indonesian population, and which was collected under the supervision of the Rand Corporation.[18] In the first wave (1993), the survey included 13 out of 27 provinces in Indonesia. These were selected based on the heterogeneity of the communities in these provinces, and included four of the five main islands in Indonesia (Sumatra, Java, Kalimantan and Sulawesi). Enumeration areas were further determined from these provinces based on the 1993 SUSENAS (National Socioeconomic Survey) sampling frame, from the 1990 census.[18–20]

Details of the complete sampling scheme and survey methods have been described in field reports.[19, 20] In brief, IFLS randomly selected 321 enumeration areas within 13 provinces, then 20 households from each urban enumeration area, and 30 households from each of the rural enumeration areas. The first survey in 1993 included over 7,000 households and more than 22,000 individuals. The second, third and fourth surveys aimed to contact all the participants from the first survey and their offspring and split-off households, with a re-contact rate of more than 90% in each subsequent wave of the households in the first wave. To date, IFLS is the only survey that provides both cross-sectional and longitudinal data in Indonesia. In this paper, each wave was treated as a separate cross sectional survey. Data from the IFLS surveys are publicly available from the Rand Corporation website [18].

Data collections for wave 1 (1993) were performed by a total of 21 field teams. Two trained team members visited every household and collected questionnaire and anthropometric data (length/ height and weight) of each household member. The length/height of children was measured using Shorr measuring boards Model 420, and weight was measured using Seca Model 770 scales (SECA, Los Angeles, CA, USA). Children who were unable to be measured alone were weighed with their parent after their parent’s weight was ascertained. Standing height was used for every child older than two years.[19] Subsequent waves applied similar methods to that of the first one.

In the IFLS survey, the information gathered from interviewees is recorded in seven separate questionnaire books.[20] We combined the household, household economy, child information, adult information and anthropometry from the four waves of datasets, resulting in 4,101 children with complete matching data for child (apart from birth weight), household and parental, and community level factors.

#### Participants

For the purposes of this study, children aged 2.0–4.9 years within each wave who had complete records for height, weight, age, and sex were selected. Even though the survey visited the same household in each wave, because waves were three to seven years apart, this resulted in a different group of 2.0–4.9 year old children in each of the waves. This is also the reason for the different number of children in each wave. For the potential risk factors analysis, we combined data from all waves.

#### Ethics

From personal communication with the IFLS research team, the IFLS survey and its procedures were reviewed and approved by Institutional Review Boards in the USA (at Rand Corporation, Santa Monica, California) and in Indonesia (Ethics Committees of Universitas Gadjah Mada, Yogyakarta, and earlier at Universitas Indonesia, Jakarta). Written informed consent was obtained from all participants. Written consent was also obtained from the next of kin, caretakers, or guardians on behalf of the children enrolled in the survey.

### Outcome variables (anthropometric calculations)

We calculated child body mass index (BMI; weight/height^2^_;_ kg/m^2^) and expressed weight, height and BMI as z-scores using age and sex specific references from the LMS Growth Program.[21] Weight, height and BMI z-scores were then calculated against the 2006 WHO Child Growth Standards for children <5 years.[17] Children with weight-for-age-z-score <-2 were categorised as underweight, those with height-for-age-z-score <-2 as stunted, and those with BMI-z-score >+1, >+2, >+3 as at risk, overweight and obese, respectively.[17, 22] The ‘at-risk’ category (BMI-z-score >+1 and ≤+2SD) was introduced by De Onis et al in 2010 for children less than five years.[22] Biologically implausible values were identified and discarded using cut off points from the WHO Anthro software (version 3.2.2, January 2011) for the Child Growth Standards (igrowup).[23]

### Potential risk factors

The conceptual framework we used was modified from the ecological model of childhood obesity, to include the potential risk factors associated with childhood malnutrition.[24] The potential risk factors for childhood malnutrition were categorised into child, parental/ household and community level factors.

#### Child factors

These consisted of the child’s age (2.0–2.9, 3.0–3.9, and 4.0–4.9 years), sex, anthropometry (birth weight [low, healthy, and high birth weight], current weight and height), and nutrition history (ever breastfed, age of weaning [full cessation of breastfeeding], and age of starting complementary foods [less or equal to/more than 6 months]).

#### Parental and household factors

These included parents’ age, marital status, anthropometry (weight and height), and maternal antenatal care history (ever/never had check-up during pregnancy). The household level factors included the parents’ education (never attended any formal education, attended primary school, middle school, and university or higher), and the household wealth index that measures the economic status of a household. The household wealth index was constructed by assigning weights to eleven household assets, including the house the family lived in, another house/building, farmland, live stock/poultry/fishpond, vehicles (cars, boats, bicycles, motorbikes), household appliances (radio, tape recorder, TV, fridge, sewing or washing machine), savings or deposits or stocks, jewellery, receivables and other assets (household furniture and utensils) using the survey data and principle components analysis method. The household wealth index was then calculated as the sum of the weighted scores for each item. The wealth index was used to rank all households across the four surveys. The household wealth index variable was categorised into five quintiles (poorest, poorer, middle, richer and richest) but for analyses in this study this index was divided into three categories. The bottom 40% of households was classified as poor households, the next 40% as the middle households and the top 20% as rich households. The complete formula and calculation of determining household wealth index have been described and used in several publications.[25–27]

For the parents, BMI was categorised using the World Health Organization International Classification of underweight, overweight, and obesity.[28] Those with heights below -2 standard deviations (SD) on the WHO 2007 Standard Growth Reference for School-aged Children and Adolescents (using the cut off points at age 19 years for male and female) were classed as having short stature.[29]

#### Community factors

Community level factors included the housing area (urban/rural) and region. The latter was classified into four, primarily based on the main Indonesian islands: Sumatra, Java, Bali and Nusa Tenggara Barat (NTB), and Kalimantan and Sulawesi.

### Statistical analysis

Data were then analysed using STATA Data Analysis and Statistical Software version 13 (STATACorp, College Station, TX).[30] The Survey (‘Svy’) command was used to adjust for clustering (enumeration areas) and sampling weights. The prevalence of underweight, stunting, at risk and overweight/obesity for each of the potential risk factors was calculated, and presented as percentage with 95% confidence intervals (CIs). Children’s weight, height and BMI within each wave were described using means and standard deviations.

To determine the associations between the potential risk factors in child, parental and community level and stunting, underweight, “at risk of overweight” and overweight/obesity in children, we combined the data from all waves. The GLLAMM (Generalised Linear Latent and Mixed Models) package with the logit link and binomial family in STATA was used to adjust for clustering and sampling weights. Univariate and multivariate binary logistic regression analysis was performed.[31] All dependent variables were categorised as dichotomous variables and odds ratios calculated. In the multivariable model, a staged modelling technique was employed.

In the first modelling stage, community level factors were first entered into the model to assess their associations with the study outcomes. A manually executed backward elimination method was conducted to select factors significantly associated with the outcomes. In the second model, the significant factors in the first stage were added to parental and household factors and this was followed by backward elimination procedure. A similar approach was used for the child factors in the third stages. A staged stepwise regression was performed because it a) avoids the degree of correlation between the important predictors; b) produces better models and a better understanding of the data; c) produces the best model (estimates) for our study; and d) avoids reporting redundant predictors. The staged stepwise regression went from the most distal set of factors (community) to the most proximal (child), because the child is a subset of the community and any future population health intervention would start from the community (more general) level and work towards the individual (more specific) level. To avoid any statistical bias, we tested and reported any collinearity in the final model. The odds ratios with 95% CIs were calculated in order to assess the adjusted risk of independent variables, and those with *P* < 0.05 were retained in the final model.

## Results

### Characteristics of participants

Table 1 shows the percentage of sample characteristics of the participants in each wave, with a total of 4,101 children aged 2.0–4.9 years. There were no significant differences in child’s age and sex across the four waves, although mean values for weight, height, and BMI increased over time. Of the 2,420 children who had a recorded birth weight, most (82.2%) had birth weight between 2.5–4.0 kg. Across all four waves, the vast majorities of children (>93%) were ever breastfed, and most (>87%) ceased being breastfed after the age of 6 months, but were given complementary food before the age of 6 months (>64%).

**Table 1:** Characteristics of children and parents in each wave of the Indonesian Family Life Survey, n (%) or mean (standard deviation).

Characteristics	Wave 1 (n = 938)	Wave 2 (n = 913)	Wave 3 (n = 939)	Wave 4 (n = 1311)
CHILD LEVEL FACTORS				
Age				
2.0–2.9	313 (33.4%)	274 (30.0%)	299 (31.8%)	425 (32.4%)
3.0–3.9	326 (34.8%)	257 (28.2%)	294 (31.3%)	454 (34.6%)
4.0–4.9	299 (31.8%)	382 (41.8%)	346 (36.9%)	432 (33.0%)
Sex				
Male	501 (53.4%)	462 (50.6%)	482 (51.3%)	633 (48.3%)
Female	437 (46.6%)	451 (49.4%)	457 (48.7%)	678 (51.7%)
Birth weight (n = 2420)				
<2.5 kg	46 (7.8%)	27 (6.1%)	19 (7.7%)	75 (6.6%)
2.5-<4.0 kg	473 (80.6%)	357 (81.1%)	198 (79.8%)	963 (84.1%)
≥4.0 kg	68 (11.6%	56 (12.8%)	31 (12.5%)	107 (9.3%)
Ever breastfed				
Yes	938 (100%)	850 (93.1%)	880 (93.7%)	1267 (96.6%)
No	0 (0%)	63 (6.9%)	59 (6.3%)	41 (3.4%)
Age of weaning				
<6 mo	41 (4.4%)	62 (6.8%)	80 (8.5%)	168 (12.8%)
≥ 6 mo	897 (95.6%)	851 (93.2%)	859 (91.5%)	1143 (87.2%)
Age starting complementary food				
<6 mo	699 (74.5%)	670 (73.4%)	720 (76.7%)	846 (64.5%)
≥ 6 mo	239 (25.5%)	243 (26.6%)	219 (23.3%)	465 (36.5%)
Child weight and height				
Weight, mean (SD)	12.5 (0.06)	12.8 (0.06)	12.9 (0.06)	13.3 (0.05)
Height, mean (SD)	91.1 (0.21)	91.8 (0.21)	91.8 (0.18)	92.8 (0.16)
BMI^a^, mean (SD)	15.1 (0.04)	15.2 (0.05)	15.2 (0.04)	15.4 (0.04)
PARENTAL AND HOUSEHOLD LEVEL FACTORS				
Mother's age				
<30 years	467 (49.8%)	368 (40.3%)	339 (36.1%)	624 (47.6%)
≥30 years	471 (50.2%)	545 (59.7%)	600 (63.9%)	687 (52.4%)
Father's age				
<30 years	217 (23.1%)	156 (17.2%)	145 (15.4%)	181 (13.8%)
≥30 years	721 (76.9%)	757 (82.8%)	794 (84.6%)	1130 (86.2%)
Parents' marital status				
Currently married	938 (100%)	913 (100%)	939 (100%)	1303 (99.4%)
Formerly married	0 (0%)	0 (0%)	0 (0%)	8 (0.6%)
Mother's BMI^b^				
Underweight	111 (11.8%)	95 (10.4%)	75 (8.0%)	121 (9.2%)
Normal weight	671 (71.5%)	636 (69.7%)	606 (64.5%)	759 (57.9%)
Overweight/obese	156 (16.7%)	182 (19.9%)	258 (27.5%)	431 (32.9%)
Father's BMI^b^				
Underweight	109 (11.6%)	105 (11.5%)	113 (12.0%)	140 (10.7%)
Normal weight	714 (76.1%)	715 (78.3%)	665 (70.8%)	905 (69.0%)
Overweight/obese	115 (12.3%)	93 (10.2%)	161 (17.2%)	266 (20.3%)
Mother's height^c^				
Normal height	417 (44.5%)	417 (45.7%)	438 (46.7%)	714 (54.5%)
Short stature	521 (55.5%)	496 (54.3%)	501 (53.3%)	597 (45.5%)
Father's height^c^				
Normal height	377 (40.2%)	376 (41.2%)	403 (42.9%)	545 (41.6%)
Short stature	561 (59.8%)	537 (58.8%)	536 (57.1%)	766 (58.4%)
Ever had check up during pregnancy				
Yes	821 (87.5%)	545 (59.7%)	653 (69.5%)	1247 (95.1%)
No	117 (12.5%)	368 (40.3%)	286 (30.5%)	64 (4.9%)
Mother's education				
No education	96 (10.2%)	58 (6.4%)	56 (6.0%)	38 (2.9%)
Primary school	508 (54.2%)	516 (56.5%)	448 (47.7%)	395 (30.1%)
Junior and high school	297 (31.7%)	285 (31.2%)	331 (35.3%)	577 (44.0%)
University or more	37 (3.9%)	54 (5.9%)	104 (11.0%)	301 (23.0%)
Father's education				
No education	70 (7.5%)	41 (4.5%)	39 (4.2%)	19 (1.5%)
Primary school	462 (49.3%)	459 (50.3%)	411 (43.8%)	262 (20.0%)
Middle school	337 (35.9%)	342 (37.5%)	357 (38.0%)	282 (21.5%)
University or more	69 (7.3%)	71 (7.7%)	132 (14.0%)	748 (57.0%)
Household's wealth index				
Poor	446 (47.6%)	801 (87.7%)	404 (43.0%)	582 (44.4%)
Middle	141 (15.0%)	69 (7.6%)	192 (20.5%)	269 (20.5%)
Rich	351 (37.4%)	43 (4.7%)	343 (36.5%)	460 (35.1%)
COMMUNITY LEVEL FACTORS				
Housing area				
Urban	435 (46.4%)	413 (45.3%)	429 (45.7%)	696 (53.1%)
Rural	503 (53.6%)	500 (54.7%)	510 (54.3%)	615 (46.9%)
Region				
Sumatra	248 (26.4%)	209 (22.9%)	206 (21.9%)	322 (24.6%)
Java	466 (49.7%)	502 (55.0%)	519 (55.3%)	647 (49.4%)
Bali & Nusa Tenggara Barat	130 (13.9%)	115 (12.6%)	103 (11.0%)	204 (15.6%)
Kalimantan & Sulawesi	94 (10.0%)	87 (9.5%)	111 (11.8%)	138 (10.4%)

SD, Standard Deviation

^a^Based upon the 2006 WHO Child Growth Standards for children <5 years [17]

^b^Based upon the WHO BMI International Classification cut-off points [28]

^c^Height below -2 Standard Deviation according to WHO Standard Growth Reference for School-aged Children and Adolescents [29]

At the parent level, most of the mothers and fathers were aged over 30 years at the time of the surveys, were currently married, and had a normal BMI. In the first three waves, just over one-half of mothers (53.3–55.5%) were classified as having short stature, whereas in wave 4, this had decreased to 45.5%. The prevalence of short stature in the fathers was higher across all four waves (57.1–59.8%). In all waves, a greater proportion of mothers ever had a check-up during their pregnancy and the majority of mothers and fathers had attended primary school or had a higher level of education.

At the community level, in all four waves there was a similar number of children living in urban and rural areas.

### Prevalence of stunting, underweight and ‘at risk of overweight’ and overweight/obesity

Table 2 shows the trends in prevalence for stunting, underweight, ‘at risk of overweight’ and overweight/obesity across the four waves, as well as the prevalence for each of the potential risk factors. The prevalence of stunting decreased significantly over 14 years–from 50.8% in 1993 (wave 1) to 36.7% in 2007 (wave 4). The same phenomenon occurred with the prevalence of underweight, which decreased significantly from 34.5% in wave 1 to 21.4% in wave 4. In contrast, the prevalence of combined at risk of overweight, overweight and obesity increased significantly over the time period, from 10.3% to 16.5%.

**Table 2:** Comparison of the prevalence of stunting, underweight and ‘at risk’ and overweight/obesity within different variables (%, 95% CIs) (n = 4,101).

Variables	Height for Age Z score <-2 (Stunted)	Weight for Age Z score <-2 (Underweight)	BMI Z score >+1 (At risk of overweight and overweight/obese)
PREVALENCE IN EACH WAVE			
Wave 1 (1993)	50.8 (47.5–53.9) ^a^	34.5 (31.6–37.7) ^a^	10.3 (8.5–12.5) ^a^
Wave 2 (1997)	48.6 (45.4–51.8)	34.6 (31.6–37.8)	10.6 (8.8–12.8)
Wave 3 (2000)	44.8 (41.7–48.0)	27.1 (24.3–30.0)	11.7 (9.8–13.9)
Wave 4 (2007)	36.7 (34.1–39.3)	21.4 (19.3–23.7)	16.5 (14.6–18.6)
CHILD LEVEL FACTORS			
Age			
2.0–2.9	48.0 (45.3–50.7) ^a^	30.4 (28.0–33.0)	16.9 (14.9–19.0) ^a^
3.0–3.9	42.5 (39.8–45.1)	26.5 (24.1–28.9)	12.5 (10.8–14.4)
4.0–4.9	43.0 (40.5–45.6)	29.1 (26.8–31.5)	9.1 (7.7–10.7)
Sex			
Male	43.7 (41.6–45.8)	27.7 (25.8–29.7)	14.4 (12.9–15.9) ^a^
Female	45.2 (43.0–47.4)	29.6 (27.7–31.6)	10.9 (9.6–12.4)
Birth weight (n = 2420)			
<2.5 kg	57.5 (49.9–64.8) ^a^	38.9 (31.8–46.5) ^a^	13.2 (8.8–19.2) ^a^
2.5-<4.0 kg	38.5 (36.4–40.6)	24.4 (22.5–26.3)	13.4 (12.0–15.0)
≥4.0 kg	33.6 (28.1–39.5)	14.9 (11.1–19.7)	21.0 (16.5–26.4)
Ever breastfed			
Yes	44.7 (42.9–46.4)	28.8 (27.3–30.4) ^a^	13.5 (12.3–14.7)
No	30.0 (17.9–45.7)	7.5 (2.4–20.8)	22.5 (12.1–37.9)
Age of weaning			
<6 mo	24.0 (19.0–29.9) ^a^	11.2 (7.7–15.9) ^a^	17.2 (12.9–22.6)
≥ 6 mo	45.3 (43.6–47.3)	29.3 (27.5–31.1)	12.9 (11.6–14.2)
Age starting complementary food			
<6 mo	43.9 (41.8–46.0)	28.3 (26.4–30.2)	13.6 (12.2–15.1)
≥ 6 mo	46.6 (43.4–49.8)	29.8 (26.9–32.8)	13.3 (11.3–15.7)
PARENTAL AND HOUSEHOLD LEVEL FACTORS			
Mother's age			
<30 years	44.1 (41.8–46.4)	29.5 (27.4–31.6)	12.9 (11.4–14.5)
≥30 years	44.7 (42.7–46.7)	28.0 (26.2–29.9)	12.6 (11.3–13.9)
Father's age			
<30 years	47.1 (43.2–51.0)	30.6 (27.1–34.3)	12.4 (10.5–14.6)
≥30 years	45.8 (44.0–47.6)	29.8 (28.1–31.5)	11.9 (10.7–13.1)
Parents' marital status			
Currently married	44.4 (42.7–46.0)	28.8 (27.6–30.1)	12.7 (11.7–13.7)
Formerly married	37.5 (12.5–71.5)	37.4 (12.5–71.5)	12.5 (1.7–53.7)
Mother's BMI ^b^			
Underweight	51.6 (46.4–56.1) ^a^	41.5 (36.8–46.4) ^a^	8.5 (6.1–11.6) ^a^
Normal	46.7 (44.8–48.6)	29.9 (28.2–31.7)	11.6 (10.4–12.8)
Overweight/obese	35.9 (33.1–38.9)	20.3 (17.9–22.8)	17.2 (15.0–19.7)
Father's BMI ^b^			
Underweight	54.4 (49.5–59.3) ^a^	41.8 (37.1–46.7) ^a^	7.6 (5.3–10.6) ^a^
Normal	48.1 (46.1–49.0)	30.5 (28.7–32.3)	12.4 (11.2–13.7)
Overweight/obese	30.5 (26.6–34.7)	18.5 (15.4–22.2)	13.2 (10.7–16.4)
Mother's height ^c^			
Normal height	32.9 (30.9–35.1) ^a^	22.0 (20.2–23.9) ^a^	11.9 (10.7–13.3)
Short stature	55.2 (53.1–57.3)	34.9 (32.9–36.9)	13.4 (12.0–14.9)
Father's height ^c^			
Normal height	35.3 (32.9–37.9) ^a^	24.4 (22.2–26.7) ^a^	11.6 (10.1–13.4)
Short stature	54.1 (51.9–56.3)	34.1 (32.1–36.2)	12.1 (10.7–13.6)
Ever had check up during pregnancy			
Yes	43.2 (41.4–44.03) ^a^	27.6 (26.0–28.2) ^a^	13.7 (12.5–15.0) ^a^
No	47.3 (44.5–50.2)	31.2 (28.6–33.9)	10.2 (8.6–12.1)
Mother's education			
No education	60.1 (53.9–66.0) ^a^	42.7 (36.7–49.0) ^a^	11.3 (7.9–15.8) ^a^
Primary school	51.9 (49.6–54.1)	34.8 (32.6–37.0)	11.9 (10.5–13.4)
Middle school	37.4 (35.0–39.9)	23.0 (20.9–25.2)	12.3 (10.7–14.1)
University or more	29.8 (26.0–34.0)	15.7 (12.8–19.2)	17.5 (14.4–21.1)
Father's education			
No education	50.3 (42.8–57.8) ^a^	40.2 (33.1–47.8) ^a^	10.1 (6.3–15.6) ^a^
Primary school	55.8 (53.4–58.3)	35.3 (32.9–37.6)	12.5 (11.0–14.2)
Junior and high school	38.9 (36.3–41.5)	26.4 (24.1–28.9)	10.6 (9.1–12.4)
University or more	32.8 (30.0–35.8)	19.3 (17.0–21.9)	16.1 (14.0–18.5)
Household's wealth index			
Poor	46.3 (44.3–48.4) ^a^	29.3 (27.5–31.2)	12.6 (11.4–14.1)
Middle	41.2 (37.4–44.2)	28.5 (25.0–32.2)	12.4 (10.1–15.3)
Rich	42.3 (39.5–44.1)	27.5 (25.0–30.1)	12.9 (11.1–14.9)
COMMUNITY LEVEL FACTORS			
Housing area			
Urban	34.9 (32.9–37.0) ^a^	23.4 (21.6–25.3) ^a^	13.0 (11.6–14.6)
Rural	53.3 (51.2–55.4)	33.5 (31.5–35.5)	12.4 (11.0–13.8)
Region			
Sumatra	49.3 (46.2–52.4) ^a^	29.5 (26.8–32.5) ^a^	11.5 (9.6–13.6) ^a^
Java	39.8 (37.3–41.8)	25.7 (23.9–27.6)	14.4 (13.0–15.9)
Bali & Nusa Tenggara Barat	52.0 (47.8–56.1)	35.5 (31.6–39.6)	10.5 (8.2–13.4)
Kalimantan & Sulawesi	46.5 (41.8–51.2)	32.6 (28.3–37.1)	9.5 (7.1–12.7)

^a^P value <0.05 (significant difference between each group within each risk factor)

^b^Based upon the WHO BMI International Classification cut-off points [28]

^c^Height below -2 Standard Deviation according to WHO Standard Growth Reference for School-aged Children and Adolescents [29]

**Fig 1:**
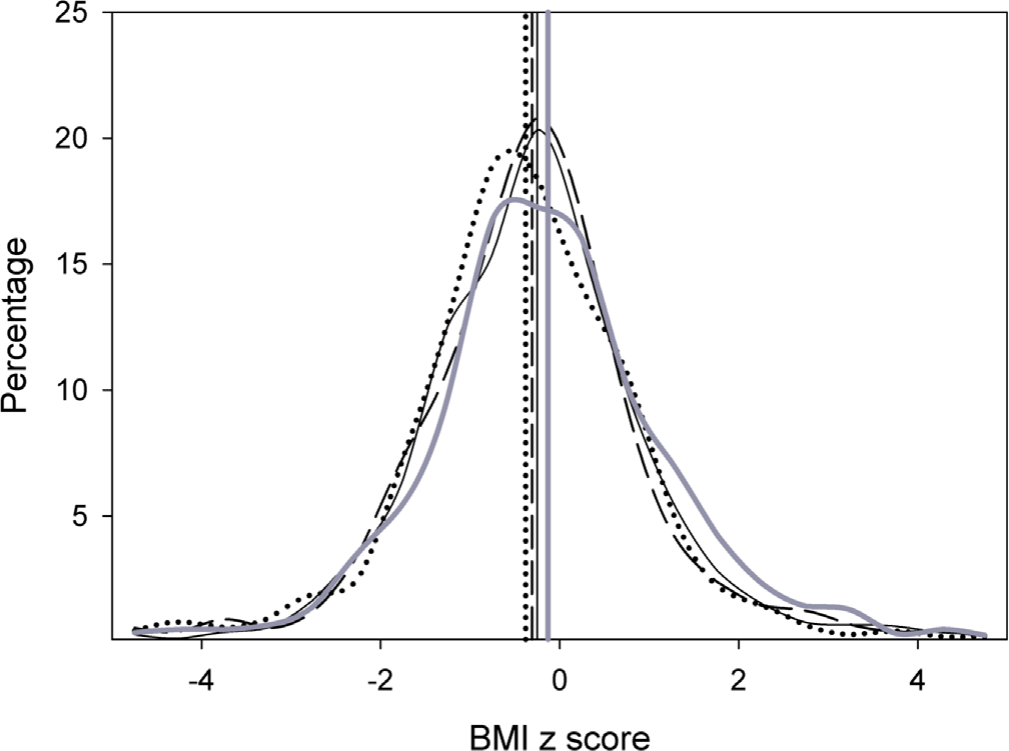
Distribution of BMI z-score and its mean (vertical line) of 2.0–4.9 years children of the Indonesian Family Life Survey (IFLS) wave 1 (1993), wave 2 (1997), wave 3 (2000), and wave 4 (2007). IFLS1: dotted line; IFLS2: dashed line; IFLS3: solid line; IFLS4: thick solid line.

Fig 1 shows the distribution of BMI-z-score for each of the four survey waves. From wave 1 to 4, there was a successive shift to the right in both the mean BMI-z-score and BMI distribution.

### Associated risk factors

Table 3 shows the unadjusted and adjusted odds ratios of all identified potential risk factors for stunting, underweight, and at risk of and overweight/obese. The multivariate analysis yielded similar results for the two forms of under nutrition: stunting and underweight. In the final model, the factors related to the higher probability of being stunted and underweight were lower birth weight (<2.5 kg), being breastfed for 6 months or more, having a mother or father who was underweight or had short stature, and mothers who never attended formal education. The only difference was that the probability of being stunted was also higher when a child lived in a rural area.

**Table 3:** Unadjusted and adjusted odds ratios (OR) (95%CI) of potential risk factor for stunting, underweight and ‘at risk’ and overweight/obesity (n = 4,101).

Variables	Height for Age Z score <-2 (Stunted)						Weight for Age Z score <-2 (Underweight)						BMI Z score >+1 (At risk of overweight and overweight/obese)					
	Unadjusted			Adjusted			Unadjusted			Adjusted			Unadjusted			Adjusted		
	OR	CI	P value	OR	CI	P value	OR	CI	P value	OR	CI	P value	OR	CI	P value	OR	CI	P value
PREVALENCE IN EACH WAVE																		
Wave 1 (1993)	Ref						Ref						Ref					
Wave 2 (1997)	0.89	0.77–1.03	0.131				0.95	0.76–1.13	0.490				1.10	0.82–1.48	0.511			
Wave 3 (2000)	0.64	0.56–0.79	<0.001				0.62	0.53–0.72	<0.001				1.17	0.87–1.56	0.288			
Wave 4 (2007)	0.49	0.41–0.56	<0.001				0.45	0.37–0.55	<0.001				1.86	1.43–2.41	<0.001			
CHILD LEVEL FACTORS																		
Age																		
2–2.9	Ref						Ref						Ref			Ref		^e^
3–3.9	0.81	0.69–0.95	0.010				0.85	0.75–0.96	0.075				0.71	0.56–0.87	<0.001	0.59	0.46–0.75	<0.001
4–4.9	0.82	0.70–0.96	0.010				0.93	0.90–1.15	0.431				0.53	0.39–0.62	<0.001	0.42	0.33–0.55	<0.001
Sex																		
Male	Ref						Ref						Ref			Ref		
Female	1.03	0.91–1.17	0.650				1.01	0.86–1.14	0.933				0.75	0.62–0.90	0.002	0.76	0.62–0.94	0.010
Birth weight																		
<2.5 kg	Ref			Ref		^c^	Ref			Ref		^d^	Ref					
2.5-<4 kg	0.54	0.38–0.73	<0.001	0.62	0.39–0.98	0.040	0.56	0.40–0.79	<0.001	0.69	0.43–1.10	0.125	0.99	0.62–1.58	0.993			
≥4 kg	0.43	0.29–0.66	<0.001	0.49	0.28–0.87	0.010	0.33	0.20–0.52	<0.001	0.39	0.21–0.74	0.004	1.47	0.85–2.54	0.158			
Ever breastfed																		
Yes	Ref						Ref						Ref					
No	0.69	0.35–1.39	0.309				0.21	0.06–0.71	0.010				2.11	0.99–4.49	0.056			
Age of weaning																		
<6 mo	Ref			Ref			Ref			Ref			Ref					
≥ 6 mo	2.73	1.96–3.14	<0.001	3.16	1.91–5.23	<0.001	2.73	2.54–6.57	<0.001	3.89	1.92–7.87	<0.001	0.78	0.53–1.15	0.217			
Age starting complementary food																		
<6 mo	Ref						Ref						Ref					
≥ 6 mo	1.05	0.90–1.24	0.475				1.04	0.89–1.24	0.588				0.99	0.81–1.27	0.864			
PARENTAL AND HOUSEHOLD LEVEL FACTORS																		
Mother's age																		
<30 years	Ref						Ref						Ref					
≥30 years	1.06	0.95–1.19	0.386				0.96	0.83–1.10	0.598				1.00	0.84–1.22	0.832			
Father's age																		
<30 years	Ref						Ref						Ref					
≥30 years	1.01	0.84–1.19	0.950				0.99	0.85–1.24	0.753				0.97	0.75–1.26	0.846			
Parents' marital status																		
Currently married	Ref						Ref						Ref					
Formerly married	0.47	0.71–3.12	0.436				1.95	0.34–11.07	0.447				0.93	0.06–12.67	0.958			
Mother's BMI^a^						^c^						^d^						^e^
Underweight	Ref			Ref			Ref			Ref			Ref			Ref		
Normal	0.79	0.64–0.97	0.020	0.71	0.48–1.00	0.050	0.59	0.48–0.74	<0.001	0.56	0.38–0.81	0.002	1.23	0.86–1.72	0.246	1.21	0.82–1.80	0.328
Overweight/obese	0.48	0.39–0.62	<0.001	0.55	0.36–0.83	0.004	0.35	0.27–0.45	<0.001	0.39	0.25–0.61	<0.001	1.85	1.23–2.67	0.001	1.88	1.24–2.87	0.003
Father's BMII^a^																		
Underweight	Ref			Ref		^c^	Ref			Ref		^d^	Ref			Ref		^e^
Normal	0.79	0.64–0.98	0.030	0.90	0.62–1.31	0.593	0.64	0.51–0.80	<0.001	0.87	0.58–1.29	0.496	1.66	1.05–2.62	0.005	1.65	1.12–2.42	0.010
Overweight/obese	0.33	0.25–0.44	<0.001	0.45	0.28–0.72	0.001	0.31	0.22–0.43	<0.001	0.53	0.32–0.90	0.020	1.72	1.18–2.51	0.020	1.49	0.09–2.38	0.092
Mother's height^b^																		
Normal height	Ref			Ref			Ref			Ref			Ref					
Short stature	2.46	2.16–2.80	<0.001	2.21	1.76–2.78	<0.001	1.77	1.54–2.04	<0.001	1.30	1.01–1.68	0.040	1.12	0.93–1.35	0.199			
Father's heightI^a^																		
Normal height	Ref			Ref			Ref			Ref			Ref					
Short stature	2.17	1.88–2.51	<0.001	1.91	1.51–2.41	<0.001	1.63	1.39–1.91	<0.001	1.49	1.15–1.94	0.002	1.07	0.87–1.33	0.472			
Ever had check up during pregnancy																		
Yes	Ref						Ref						Ref					
No	1.10	0.96–1.26	0.167				1.17	1.01–1.36	0.030				0.72	0.58–1.12	0.004			
Mother's education																		
No education	Ref			Ref		^c^	Ref			Ref		^d^	Ref					
Primary school	0.77	0.58–1.00	0.050	0.92	0.49–1.75	0.822	0.69	0.52–0.90	0.008	0.76	0.40–1.44	0.411	0.92	0.62–1.37	0.688			
Middle school	0.37	0.28–0.49	<0.001	0.47	0.24–0.91	0.020	0.34	0.26–0.46	<0.001	0.36	0.19–0.70	0.003	0.99	0.66–1.49	0.985			
University or more	0.31	0.22–0.42	<0.001	0.59	0.29–1.19	0.144	0.23	0.16–0.33	<0.001	0.28	0.13–0.59	0.001	1.60	1.03–2.48	0.030			
Father's education																		
No education	Ref						Ref						Ref			Ref		^e^
Primary school	1.32	0.97–1.80	0.070				0.73	0.53–1.00	0.050				1.68	0.98–2.90	0.058	1.78	1.01–3.17	0.040
Junior and high school	0.60	0.44–0.82	0.002				0.45	0.32–0.62	<0.001				1.33	0.76–2.32	0.310	1.33	0.74–2.41	0.332
University or more	0.47	0.34–0.65	<0.001				0.31	0.22–0.44	<0.001				2.22	1.28–3.86	0.004	2.19	1.17–4.11	0.010
Household's wealth index																		
Poor	Ref						Ref						Ref					
Middle	0.82	0.68–0.95	0.040				0.98	0.81–1.21	0.966				0.94	0.72–1.23	0.683			
Rich	0.79	0.69–0.92	0.003				0.91	0.78–1.07	0.295				1.01	0.80–1.23	1.000			
COMMUNITY LEVEL FACTORS																		
Housing area																		
Urban	Ref			Ref			Ref						Ref					
Rural	2.23	1.95–2.55	<0.001	1.55	1.22–1.97	<0.001	1.77	1.52–2.05	<0.001				0.91	0.75–1.10	0.338			
Region																		
Sumatra	Ref						Ref						Ref					
Java	0.69	0.53–0.90	0.007				0.75	0.54–1.04	0.089				1.27	1.06–1.50	0.049			
Bali & Nusa Tenggara Barat	1.06	0.65–1.71	0.804				1.12	0.70–1.80	0.617				0.80	0.50–1.26	0.343			
Kalimantan & Sulawesi	0.88	0.52–1.49	0.650				1.08	0.66–1.77	0.750				0.73	0.45–1.18	0.203			

^a^Based upon the WHO BMI International Classification cut-off points [28]

^b^Height below -2 Standard Deviation according to WHO Standard Growth Reference for School-aged Children and Adolescents [29]

^c^ *P*-value for the whole category: (Birth weight, P-value = 0.047); (Mother’s BMI, P-value = 0.017); (Father’s BMI, P-value <0.001); (Mother’s education, P-value <0.001)

^d^ *P*-value for the whole category: (Birth weight, P-value = 0.013); (Mother’s BMI, P-value <0.001); (Father’s BMI, P-value = 0.035); (Mother’s education, P-value <0.001)

^e^ *P*-value for the whole category: (Age, P-value <0.001); (Mother’s BMI, P-value <0.001); (Father’s BMI, P-value = 0.034); (Father’s education, P-value = 0.005)

Children had a greater probability of being at risk, or overweight/obese when they were in the youngest age group (2–2·9 years), were male, or had a mother and father who were overweight/obese or had fathers who had attended university.

## Discussion

### Statement of principal findings

Our study, a secondary analysis of a series of representative surveys in Indonesian children aged 2.0–4.9 years, showed that, between 1993 and 2007, there was a significant decrease in the prevalence of both stunting and underweight. Over the same period there was a significant increase in the prevalence of ‘at risk of overweight’ and overweight/obesity in this age group.

The issues of underweight and stunting are points of focus for Indonesian health authorities. Strategies to tackle them have included a variety of nutrition programs[32–34] as well as major improvements in the delivery of health care services, hygiene, and sanitation, including access to clean water.[33] These different approaches help explain the decrease in the prevalence of both underweight (from 34.5% to 21.4%) and stunting (from 50.8% to 36.7%) in children aged 2.0–4.9 years and over the 14 year period of the survey.

The exact reason behind the rising prevalence of at risk/ overweight/ obesity in young children in Indonesia is not known. However, de Onis et al suggested that the rising global prevalence of overweight/obesity in early childhood resulted from changes in both nutrition and physical activity patterns.[22] For example, improvements in the economy have resulted in changes in active transport and incidental activity[35] as well as a shift in dietary intake.[33, 36]

Like many other developing countries, education is an important issue for Indonesia. Across the four waves, the level of education improved for both sexes, with increasing numbers of mothers and fathers experiencing formal education. The near doubling of the percentage of university-graduate parents is seen in wave 2 to 3, and from wave 3 to 4, the percentage of mothers with university education more than doubled. In families with limited income, the culture in many Asian countries still influences parents to choose their boys over the girls to go to university, because they will become the breadwinner for the family. This is supported by the higher numbers of men, compared to women, who graduated from university in all four waves.

The percentage of poor households in wave 2 was much higher than in the other waves (87.7% vs. 47.6% in wave 1, 43.0%, in wave 3, 44.4% in wave 4). This might be an effect of the Asian financial crisis, which happened in 1997 (around wave 2 of the survey, conducted from late 1997 to 1998). Indonesia was one of the countries most affected by the crisis. At that time, many families had a dramatic decrease in family income as the inflation rate was 80% in that year.[37]

Our study indicates that the “double burden of malnutrition” is present in Indonesian children aged 2.0–4.9 years. We also identified that both stunting and underweight were associated with a lower birth weight, being breastfed for more than 6 months, indices of parental under nutrition, and lack of maternal formal education. In contrast, being “at risk of overweight/obesity” or overweight or obese was associated with being in the youngest age group (2.0–2.9 years), being male, parental over nutrition, and high paternal formal education.

### Strengths and limitations

This study is the first to elaborate on the temporal trends in, and potential risk factors for, the three major forms of malnutrition in Indonesian children aged 2.0–4.9 years: stunting, underweight, and overweight/obesity. Another strength includes the use of the category of ‘at risk of overweight’ i.e. children with BMI-z-score >+1SD and ≤+2.[22] In addition, analyses are based on data derived from a representative sample of the Indonesian population with the use of sampling weights in the analysis to reduce bias, and measurements were performed by trained professionals. To handle missing values in the birth weight variable in our dataset, we performed multiple imputations and the results show no differences between the complete data and the 5 and 10 imputation data sets (S1 Table). One limitation is the use of repeated cross sectional surveys, which does not allow us to infer causality. In addition, the risk factor analyses were limited: we were not able to investigate all potential risk factors, such as parental occupation or health knowledge, due to insufficient data or the question not being addressed in the questionnaire.

### Comparison with other studies

Several studies using different data sources in Indonesia have yielded comparable prevalence results to our study, although all were performed in children aged 0–5 years and usually focused on one type of malnutrition.[15, 38–40] None has previously identified the presence of the double burden of malnutrition in this age group (2.0–4.9 years). We opted to use the age of 2.0 years as the lower age limit in this study to ensure the process of stunting was fully developed in these children, making this study different from any previously published studies. However, both under nutrition (stunting or underweight) and concurrent overweight/obesity have been documented in populations of under-five children in several Asian and Latin America countries such as Malaysia, Vietnam, China, Nepal, Ecuador, Mexico, Guatemala, and Colombia.[36, 41–46] Furthermore, studies of temporal trends in such countries as Mexico and Colombia show the same phenomenon of decreasing prevalence of stunting and an increasing prevalence in overweight/obesity.[43, 46]

The prevalence of stunting in 2.0–4.9 Indonesian children decreased over the period of the four surveys, although it was still considered to be high, at 36.7% in the most recent wave of the study in 2007. A 2013 review showed wide-ranging prevalence rates of stunting in under five children, such as 23.3% in Vietnam, 32.3% in the Philippines, 35.1% in Myanmar, 44.2% in Ethiopia, and 59.3 in Afghanistan.[47] A Malaysian study showed a 14.2% prevalence of stunting in children aged 1.0–3.9 years in 2013.[44]

The prevalence of underweight in this age group in Indonesia also decreased over the period of the survey, to 21.4% at wave 4. A recent review of the prevalence of moderate to severe underweight in children under five in Asian countries showed marked variations in prevalence, ranging from 8.0% in China, 11.0% in Malaysia, 18.0% in Thailand, 28.0% in the Philippines, to 47.0% in India and 48.0% in both Nepal and Bangladesh.[36] The prevalence of underweight in under-five children in Mexico, Guatemala, and Colombia are 2.8%, 1.0%, and 3.4%, respectively.[43, 45, 46]

The prevalence of at risk/overweight/obesity in wave 4 was 16.5%, lower than in Vietnam 2005, where 36.8% of 4.0–5.0 year old children were overweight/obese.[41] The prevalence of at risk/ overweight/ obesity in under five children in Ecuador in 2012 was 30.2%[42] and in under five children in Mexico and Guatemala was 9.0% and 4.9%, respectively.[43]

However, prevalence rates are age specific, and the definition/cut offs used for underweight and overweight/obesity in different studies might be different, highlighting the need for caution in making direct comparisons between studies.

Our findings are consistent with other research showing strong associations between stunting and a lower birth weight,[38, 48, 49] a longer duration of breastfeeding,[49] short-statured mothers,[11, 38, 48] underweight mothers,[48] less educated mothers,[11, 38, 48–50] and living in rural areas.[38] Our study also emphasised the association between underweight children and mothers’ and fathers’ education levels.[40] Likewise, we found an association between overweight/obesity and male sex,[11] and maternal overweight/obesity.[11, 48]

### Implications for research, policy and practice

Our study highlights the emerging issue of the double burden of malnutrition in young Indonesian children. Many interventions and strategies that are already in place for the management of under nutrition in Indonesia, e.g. the Healthy and Fit due to Balance Nutrition (Sehat dan Bugar berkat Gizi Seimbang) or the Scaling Up Nutrition movement,[32, 34] may need to be modified to respond to the problem by balancing the risk factors associated to each condition. For example, interventions that aim to correct under nutrition in early life need to emphasise the importance of *both* linear growth *and* appropriate weight.[33, 51–53] Fortification of complementary foods needs to be balanced so as not to make previously ‘healthy weight’ children become overweight/obese. Further interventions in school age children need to balance the promotion of healthy diets as well as physical activity.

Another important point is to ensure that interventions start as early as possible. For example, improvements in diets should start with adolescent girls and young women in their pre-pregnancy state, in order to prevent having underweight mothers, which is in turn a risk factor for having stunted and/ underweight children. The fact that breastfeeding decreases the prevalence of obesity in later life is irrefutable;[54] however, prolonged breastfeeding, in association with poor feeding practices may be associated with stunting.[49, 55] Therefore, parental education, especially of mothers, regarding the importance of breastfeeding combined with healthy feeding practices is important.

Currently, these different conditions of malnutrition are treated as separate issues.[33] There should be a policy that combines the management of concurrent under and over nutrition. Future studies should also aim at exploring whether children who are stunted in early life are more likely to be overweight/obese in later life.

## Supporting Information

S1 Table Adjusted odds ratios (95% confidence intervals) for the complete data, 5 and 10 imputation data sets (M = 5 and M = 10, respectively). (DOCX)
