# Supplementary material for: Stunting, Underweight and Overweight in Children Aged 2.0–4.9 Years in Indonesia: Prevalence Trends and Associated Risk Factors
Source: PLoS One. 2016 May 11;11(5):e0154756. doi: 10.1371/journal.pone.0154756 (PMC4864317; doi:10.1371/journal.pone.0154756)
Supplement: S1 Table — (DOCX) [file pone.0154756.s001.docx]

# S1 Table. Adjusted odds ratios (95% confidence intervals) for the complete data, 5 and 10 imputation data sets (M=5 and M=10)

| Variables | Complete data | | | | M=5 | | | | M=10 | | | |
| --- | --- | --- | --- | --- | --- | --- | --- | --- | --- | --- | --- | --- |
|  | AOR | [95% CI] | | P>z | AOR | [95% CI] | | P>t | AOR | [95% CI] | | P>t |
| Housing area | 1.62 | 1.28 | 2.06 | 0.000 | 1.71 | 1.38 | 2.12 | 0.000 | 1.70 | 1.38 | 2.11 | 0.000 |
| Mother’s BMI | 0.77 | 0.64 | 0.94 | 0.010 | 0.77 | 0.63 | 0.92 | 0.005 | 0.77 | 0.63 | 0.92 | 0.005 |
| Fathers’s BMI | 0.65 | 0.51 | 0.81 | 0.000 | 0.62 | 0.50 | 0.78 | 0.000 | 0.62 | 0.50 | 0.78 | 0.000 |
| Mother’s height | 2.23 | 1.78 | 2.80 | 0.000 | 2.14 | 1.73 | 2.64 | 0.000 | 2.14 | 1.73 | 2.64 | 0.000 |
| Father’s height | 1.95 | 1.54 | 2.45 | 0.000 | 1.89 | 1.53 | 2.34 | 0.000 | 1.89 | 1.53 | 2.35 | 0.000 |
| Mother’s education | 0.71 | 0.60 | 0.83 | 0.000 | 0.71 | 0.61 | 0.82 | 0.000 | 0.71 | 0.61 | 0.82 | 0.000 |
| Birth weight | 0.72 | 0.55 | 0.96 | 0.023 | 0.79 | 0.60 | 1.03 | 0.082 | 0.81 | 0.62 | 1.06 | 0.117 |
| Age of weaning | 3.07 | 1.87 | 5.05 | 0.000 | 2.69 | 1.73 | 4.19 | 0.000 | 2.69 | 1.72 | 4.18 | 0.000 |
